# Factors associated with early resumption of sexual intercourse among women during extended postpartum period in Northwest Ethiopia: a cross sectional study

**DOI:** 10.1186/s40834-020-00124-7

**Published:** 2020-11-02

**Authors:** Birye Dessalegn Mekonnen

**Affiliations:** Department of Nursing, Teda health Science College, P.O.BOX: 790 Gondar, Ethiopia

**Keywords:** Early resumption of sexual intercourse, Postpartum women, Ethiopia

## Abstract

**Background:**

Postpartum sexual health and practice need to be integrated in the current maternal healthcare services to address sexual health problems. However, postpartum sexual practice has received little attention, and was not often discussed by healthcare providers during prenatal and postnatal care. Thus, this study was aimed to assess early resumption of sexual intercourse and associated factors among postpartum women in Gondar city, Northwest Ethiopia.

**Methods:**

A community based cross-sectional study was conducted from January 20 to February 20, 2020. A systematic random sampling technique was used to select 634 postpartum women. A pretested, structured and interviewer-administered questionnaire was used to collect data. Data were entered into Epi Info 7.2.2 and exported to SPSS version 20 for analysis. Bivariable and multivariable logistic regressions analysis were done. Variables with *p*- value of < 0.05 were considered as statistically significant.

**Results:**

The magnitude of early resumption of sexual intercourse after childbirth was found to be 26.9% (95% CI: 23.2, 30.8). Urban resident (AOR = 6.12, 95% CI: 2.41, 15.66), parity of one (AOR = 2.26, 95% CI: 1.66, 7.78), husband demand (AOR = 2.66, 95% CI: 1.72, 4.11), postnatal care (AOR = 1.45, 95% CI: 1.06, 2.18) and use of family planning (AOR = 2.72, 95% CI: 1.51, 3.43) were factors significantly associated with early resumption of sexual intercourse.

**Conclusion:**

The study found that more than one fourth of women had resumed sexual intercourse within six weeks of following childbirth. The finding of this study suggests the need of integrating discussions of postpartum sexual activity into routine prenatal, intrapartum and postnatal care with collaborative effort of policy makers, program planners, health care providers and other stakeholders. Moreover, spousal communication on postpartum sexual activity should be encouraged.

## Background

During the first weeks after giving birth women face physical, psychological, social, and emotional challenges as well as changes in sexual function practices [1]. Postpartum period is the period beginning immediately after the birth of the child, and it is an ideal time to deliver interventions that improve the health and survival of both the newborn and the mother [2]. Although childbirth bring many changes to the postpartum sexual health and well-being of mothers, a period following childbirth has great expectation for parents as they look forward to getting new healthy baby [3].

Sexual practice during the postpartum period is an important element that has been identified in women’s healthcare which needs increasing attention worldwide [4]. According to the World Health Organization (WHO) recommendations, as part of an overall assessment following childbirth all women should be assessed regarding resumption of sexual intercourse and possible dyspareunia [5]. However, in developing countries, health promotion and education on postpartum sexual health is limited in current maternal health care services [6, 7]. Thus**,** substantial proportion of postpartum women do not receive information or advice about when to resume sexual activity after birth [5].

Interest in sexual activity often decrease for the period of pregnancy, but eventually return to normal during postpartum period [8]. In many countries of sub-Saharan Africa, sexual intercourse after birth is sensitive issue and considered a taboo, and sexual abstinence after childbirth is a common cultural practice [9, 10].

Initiation of sexual intercourse early in the postpartum period may expose women at risk of getting pregnant, which is most likely unintended. Though more than 90% of women during the postpartum period want to either delay or avoid future pregnancies globally, women usually resume sexual activity without use of any modern contraceptives [11]. Though the resumption of sexual activity following childbirth varies from culture to culture, WHO recommends that women should wait till 24 months after childbirth, before becoming pregnant again [12].

Even though there is limited information on factors that affect women’s early return to sexual activity during the postpartum period, biological, psychological, sociological and religious practice, educational status and parity of the women were identified factors that can affect women’s decision on their sexual practice within the postpartum period [8, 13–15].

Women may have problems with sexual function and relationship in their postnatal period, for which they may require adequate information, counseling and support to be prepared for resumption of sexual intercourse in the postpartum, and to avoid complications such as puerperal infection and unwanted pregnancies [14]. Since, postpartum sexual health is inseparable from sexuality and sexual function, perceived sexual problems in the postpartum period need to be addressed [7, 16]. However, postpartum sexual practice has received little attention with policy makers, programmers and other stakeholders, and was not often discussed by health care providers during prenatal and postnatal care [17, 18]. For the effective intervention of postpartum sexual health, determine the magnitude of early sexual resumption and understanding the sexual experiences of women during the postpartum period is important. Therefore, the aim of this study was to assess the early resumption of sexual intercourse after childbirth and associated factors among women in the extended postpartum period in Gondar city, Northwest Ethiopia. This study can help policy makers, health care professionals and other stakeholders to provide information on postpartum sexual practice to women and their partners during antenatal care and after childbirth.

## Methods

### Study design, period and area

A community-based cross-sectional study was conducted from January 20, 2020 to February 20, 2020 at Gondar city. Gondar city is located 727 KM Northwest of Addis Ababa, capital of Ethiopia. It is divided in to seven sub cities and 25 kebeles. According to the 2013 population projection estimate, there were 258,178 residents and more than half of them were females [19]. The city has one comprehensive specialized hospital, eight governmental health centers and 14 rural health posts that are providing maternal and other health services to the population.

### Source and study population

The source population was all women of child bearing age who gave birth in the past 12 months prior to the study period in Gondar city, northwest Ethiopia. The study participants were women who gave birth in the past 12 months prior to the study and lived at least for 6 months in the selected Kebeles, but those who were sick and unable to respond during study period were excluded.

### Sample size determination and sampling procedure

The sample size was determined using single population proportion formula considering the following assumptions: expected proportion of women who resumed sexual intercourse within the six weeks postpartum of 50%; marginal error of 5, 95% confidence level, design effect 1.5 and estimated non response rate of 10%. Finally, the total sample size calculated was 634.

A multistage cluster sampling technique was used to identify the participants. First, out of the six sub-cities of Gondar city, Maraki sub-city was chosen by the simple random sampling technique. From Maraki sub-city, four kebeles (College mazoria, Samuna ber, Shiro meda and Hidassie) were selected using the lottery method. Then, the total sample size was allocated proportionally to each selected kebeles. The study subjects were selected by systematic random sampling. The sampling interval was 2 obtained by dividing the study population to the sample size (1167/634 ≈ 2). The first household was chosen at the center of each Keble by lottery method as a starting point, and then the data collectors were going in the right direction until the required sample size was achieved. If there were more than one eligible woman in the selected household, one was considered by lottery method for the interview.

### Data collection tool and procedure

Structured and pretested questionnaire adapted from related literatures [8, 13, 20–23] was used. Data were collected through face-to-face interview at the participant’s home. The questionnaire was first developed in English, then translated into Amharic, and finally retranslated into English to check the consistency. Four diploma female midwives as data collectors and two supervisors were involved in the data collection process. Training was given to the data collectors and supervisor. The collected data were checked for completeness daily.

### Variables of the study

In this study, the dependent variable was the early resumption of postpartum sexual intercourse (before or after six weeks postpartum), which is defined as having the first penetrative sexual intercourse after childbirth. The timing of sexual resumption was categorized into resumption before 6 weeks (early resumption) and after 6 weeks (recommended resumption). The independent variables include marital status; age, religion, use of contraceptive, place of residence, monthly income, woman’s education, partner’s education, mode of delivery, total child ever born, current working status of respondent, breastfeeding, menstrual resumption, family planning use, antenatal care (ANC), place of delivery and PNC.

### Data quality control

Training was given to the data collectors and supervisor on general objective of the study, contents of the tool, how to approach the study participants and keep their confidentiality. The tool was pre-tested on 5% (32 women) of the sample in Debark town which was out of the study area before starting actual data collection.

### Data processing and analysis

Data were entered using EPI-INFO version 7.2.2.2 and exported to Statistical Package for Social Sciences (SPSS) version 20 for further analysis. Descriptive statistics were computed to describe the study population in relation to relevant variables. For the wealth index, a principal component analysis was conducted. Both bivariable and multivariable logistic regression model were carried out to identify associated factors. Variables with *p*-values of < 0.2 in the bivariate analysis were further fitted to multivariable logistic regression analysis. Adjusted Odds Ratio (AOR) with 95% confidence intervals (CI) was computed to determine the strength and presence of association. Variance inflation factors (VIF) was used to check assumptions regression model, and model goodness of fit was tested by the Hosmer–Lemeshow model goodness-of-fit test. Finally, variables with p- values of < 0.05 were considered as statistically significant.

### Ethical approval

Ethical clearance was obtained from the Institutional Review Board (IRB) of University of Gondar, College of Medicine and Health Sciences, Institute of Public Health. An official letter of cooperation was written to Gondar city administration. The benefit of the study was explained to the respondents, and then verbal informed consent was received from the participants before interviewing them. Confidentiality of the information was maintained by excluding personal identifiers in the questionnaire.

## Results

### Socio-demographic characteristics of study participants

In this study, a total of 634 study participants were responded the interviewed with a response rate of 100%. The mean age of the respondents was 28.3 (SD ±5.5) with the minimum and the maximum age of 18 and 50 years respectively. Majority, 611(89.5%) of the participants were married and about, 546(86.6%) of women were urban residents. Five hundred forty-six (86.2%) of participants were followers of Orthodox Christian. Regarding to educational status, 224(35.3%) of women had completed secondary school while 267(43.7%) of their husband had completed higher education (Table 1).

**Table 1:** Socio-demographic characteristics of mothers in the extended postpartum period, Gondar city, northwest Ethiopia, 2020 (*n* = 634)

Variables	Number	Percent
Age		
18–24	205	32.3
25–34	331	52.2
> 35	98	15.5
Marital status		
Married	611	96.4
Unmarried (in union)	23	3.6
Religion		
Orthodox	546	86.2
Muslim	82	12.9
Protestant	6	0.9
Residence		
Urban	546	86.2
Rural	88	13.8
Educational level		
No formal education	116	18.3
Primary school complete	105	16.6
Secondary school complete	224	35.3
Higher education complete	189	29.8
Occupation		
Housewife	416	65.6
Government employee	70	11.0
Private employee	112	17.7
Merchant	19	3.0
Others (Daily laborer, student, Tella sellers)	17	2.7
Husband’s education status (*N* = 611)		
No formal education complete	92	15.1
Primary school complete	88	14.4
Secondary school complete	164	26.8
Higher education complete	267	43.7
Husband’s occupation (*N* = 611)		
Farmer	41	6.7
Government employee	198	32.4
Private employee	165	27.0
Merchant	131	21.4
Daily laborer	59	9.7
Others *(Driver, student, job seekers)*	17	2.8
Monthly income		
Lowest	64	10.1
Second	191	30.1
Middle	101	15.9
Fourth	142	22.4
Highest	136	21.5

### Obstetrical and sexual history of participants

The median number of living children was 2 per women (IQR = 1.0 3.0) with nearly, 304(47.9%) of women had two to three living children. Majority, 389 (61.4%) of participants had vaginal delivery with no episiotomy/tears, and 325 (51.2%) of their babies were aged between 6 and 24 weeks old.

Among the participants, 569 (89.7%) had already resumed sexual intercourse after delivery. Of the participants who had resumed sexual intercourse, 153(26.9%) with 95% CI (23.2, 30.8) resumed before 6 weeks after childbirth and 416 (73.1%) resumed after 6 weeks. The timing of resumption of sexual intercourse ranged between 2 and 46 weeks with a median of 12 weeks. The major reason (76.1%) for resumed sexual intercourse after delivery were partner demand. In relation to problems noted after resuming sexual intercourse, about 394(69.2%) of participants had no problems experienced and 89(15.6%) had experienced pain during sexual intercourse.

Concerning maternal healthcare service utilization about 617(97.3%) of the respondents had antenatal attendance, about 577 (91.0%) of women delivered their last child at public health facilities and 347(54.7%) attended postnatal clinic after their last delivery. More than half, 329(51.9%) women were using a family planning method. Of the participants who use family planning method, only 56(17.1%) were initiated by 6 weeks following childbirth (Table 2).

**Table 2:** Reproductive and obstetric characteristics of mothers of extended postpartum period in Gondar city, northwest Ethiopia, 2020 (*n* = 634)

Variables	Number	Percent
Parity		
1	227	35.8
2–4	373	58.8
> 5	34	5.4
Alive children by now		
1	232	36.6
2–3	304	47.9
> 4	98	15.5
Was the last pregnancy planned?		
Yes	593	93.5
No	41	6.5
Mode of delivery for current child		
Vaginal without episiotomy/tear	389	61.4
Vaginal with episiotomy/tear	75	11.8
Instrumental (Forceps and vacuumed delivery)	41	6.5
Emergency Cesarean section	89	14.0
Elective Cesarean section	40	6.3
Breastfeed currently		
Yes	614	96.8
No	20	3.2
Sexual intercourse resumed after recent birth		
Yes	569	89.7
No	65	10.3
Timing of resumption sexual intercourse (*n* = 569)		
Before 6 weeks	153	26.9
After 6 weeks	416	73.1
Problems noted after resuming sexual intercourse (*n* = 569)		
No problem	394	69.2
Pain during sex	89	15.6
Vaginal bleeding/discharge	34	6.0
Lack of desire	29	5.1
Vaginal dryness	23	4.0
Did you seek medical advice for sexual problem (*n* = 175)?		
Yes	52	29.7
No	123	70.3
Reason for resuming sexual intercourse		
I wanted sex	136	23.9
Partner demand	433	76.1
Ever advised about postpartum sexual activity		
Yes	181	28.5
No	453	71.5
Time from childbirth		
Less than 6 weeks	36	5.7
6–24 weeks	325	51.2
24–46 weeks	273	43.1
Using any family planning		
Yes	329	51.9
No	305	48.1
Have ANC follow up		
Yes	617	97.3
No	17	2.7
Place of delivery		
Public health facility	577	91.0
Private clinics	13	2.1
Home	44	6.9
Postnatal care		
Yes	347	54.7
No	287	45.3

### Factors associated with early resumption of sexual intercourse after delivery

Variables that had *p* values < 0.2 in the bivariate analysis were included in the multivariable logistic regression model. In the multivariable logistic regression analysis, place of residence, parity, husband demand for sexual intercourse, use of family planning, and PNC visit were significantly associated with early resumption of sexual intercourse after childbirth. Women living in urban setting were 6.12 times (AOR = 6.12, 95% CI: 2.41, 15.66) more likely to initiate early sexual intercourse after childbirth than those who live in rural areas. The odds of early resumption of sexual intercourse after childbirth among women with one parity were about 2.26 times higher than women with five and above parity (AOR = 2.26, 95% CI: 1.66, 7.78). The odds of early sexual intercourse resumption after childbirth in husband demand for sexual intercourse were about 2.66 times higher than women’s desire (AOR = 2.66, 95% CI: 1.72, 4.11). Women who obtained PNC were about 1.45 times higher odds to initiate early sexual intercourse than who did not (AOR = 1.45, 95% CI: 1.06, 2.18). Women who were using family planning were about 2.72 times higher odds to resume early sexual intercourse (AOR = 2.72, 95% CI: 1.51, 3.43) than those who did not use (Table 3).

**Table 3:** Bivariable and multivariable analysis for early resumption of sexual intercourse among mothers of extended postpartum period in Gondar city, northwest Ethiopia, 2020

Variables	Timing of resumption		COR (95% CI)	AOR (95% CI)
	Before 6 weeks	After 6 weeks		
Residence				
Urban	146	352	3.79(1.18, 5.89)	**6.12(2.41, 15.66)**
**Rural**	**7**	**64**	**1**	**1**
Age				
18–24	49	138	0.97(0.54, 1.72)	0.92(0.43, 1.99)
25–34	82	214	0.90(0.52, 1.55)	0.99(0.52, 1.86)
> 35	22	64	1	1
Educational status				
No formal education	34	64	0.53(0.11, 0.92)	0.54(0.29, 1.02)
Primary school complete	30	57	0.54(0.31, 0.95)	0.49(0.26, 1.94)
Secondary school complete	49	154	0.89(0.55, 1.44)	0.88(0.52, 1.48)
College/University complete	40	141	1	1
Parity				
1	46	153	1.57(0.64, 3.86)	**2.26(1.66, 7.78)**
2–4	99	246	1.17(0.49, 2.80)	1.81(0.59, 5.54)
> 5	8	17	1	1
Breastfeed currently				
Yes	149	404	0.91(0.29, 2.85)	1.01(0.29, 3.48)
No	4	12	1	1
Reason for resuming sexual intercourse				
I wanted sex	59	77	1	1
Partner demand	94	339	2.76(1.82, 4.16)	**2.66(1.72, 4.11)**
Ever advised about postpartum sexual activity				
Yes	35	109	1.20(0.77, 1.85)	1.32(0.79, 2.19)
No	118	307	1	1
Using any family planning				
Yes	65	250	2.04(1.40, 2.97)	**2.72(1.51, 3.43)**
No	88	166	1	1
Have postnatal care				
Yes	69	244	1.73(1.19, 2.51)	**1.45(1.06, 2.18)**
No	84	172	1	1

## Discussion

The objectives of this study was to assess the timing of sexual intercourse resumption after childbirth and identify factors associated with early resumption of sexual intercourse among postnatal women. Accordingly, the finding shows that more than one fourth of women had resumed sexual intercourse by 6 weeks of postpartum. This implies that substantial proportion of women could be at risk of unintended pregnancy, especially when they delay to initiate postpartum contraceptive methods or intensity of breastfeeding could not be guaranteed. Evidence revealed that short birth interval is relatively rare when sexual activity is delayed in the postpartum [9]. Furthermore, sexual abstinence is beneficial to the mother, for the body’s recovery and the process of healing the birth injuries [24, 25].

In this study, the magnitude of early sexual intercourse resumption was found to be 26.9% (95% CI: 23.2, 30.8). This finding is consistent with similar studies done in Nigeria [26] and Ghana [15], which reported early sexual intercourse resumption as 29.7 and 23.9% respectively. However, this finding was higher than the findings of similar studies previously done in Uganda (21.9%) [21] and Northern Nigeria 21.7% [27]. However, this finding was found to be lower when compared with study done in United States (43%) [28], Nigeria (38.9%) [20], and Iban (37.4%) [8]. The possible explanation for these variations could be due to differences in socio-cultural, social beliefs and norms, religious practices, and sexual attitudes of women in different parts of the world. Early sexual resumption is influenced by subjective norms, social beliefs, and values held by society about post-partum sexual abstinence [29].

The present study revealed that women who live in urban setting were 6.12 times (AOR = 6.12, 95% CI: 2.41, 15.66) more likely to resume early sexual intercourse after childbirth than rural dwellers. This finding is consistent with other studies done in Nigeria [22] and Eswatini [24]. This could be due to the fact that women living in rural area are more likely to adhere to traditional practices, and the cultural practice of sexual abstinence during the postpartum period. In addition, most women in rural residents separated from their spouses or partners and stayed with parents or relatives during the postpartum period [24, 30].

This study was also identified that husband demand for sexual intercourse were about 2.66 times higher than women’s desire (AOR = 2.66 95% CI: 1.72, 4.11) to early resumption of sexual intercourse after childbirth. This finding is supported by other studies done in Eswatini [24] and Tanzania [30]. This attributed to the male partner’s demand and pressure for sexual intercourse. In African, women usually initiate sexual intercourse against their will due to fear that the men will go elsewhere to have sex with other women [24, 27, 31]. Furthermore, the finding implies that the need of routine counseling and encouraging of couples, especially women, to voice out sexual concerns during the postpartum period. This could be achieved with the effort of health care providers through adopting existing guidelines to local cultural sensitivities and individual preferences [32].

Women with one parity were about 2.26 times higher (AOR = 2.26, 95% CI: 1.66, 7.78) to resume early sexual intercourse after childbirth than women with five and above parity. This finding is in agreement with a study conducted in Uganda [21]. The possible explanation for this might be that women might need another child within a short time interval, so that they couldn’t assume that they are at risk of getting pregnancy.

Women who obtained PNC were about 1.45 times higher odds to initiate early sexual intercourse than who did not (AOR = 1.45, 95% CI: 1.06, 2.18). This might be due to that postnatal care visit could give the opportunity of getting more information and counselling from health professionals on exclusive breast feeding and postpartum contraceptive use, so that they can initiate postpartum contraception on recommended time. Studies reported contraceptive use as one of the factors associated with early postpartum resumption of sexual intercourse [21, 23, 33]. Furthermore, women who obtained PNC could get more information on sexual practice following delivery including misconceptions and can help women to practice sexual intercourse with no fear of physiological changes.

Women who were using family planning were about 2.72 times higher odds to resume early sexual intercourse (AOR = 2.72, 95% CI: 1.51, 3.43) than those who did not use. This finding is consistent with other previous studies done in rural Uganda [34], Iban [8] and Uganda [21]. This finding could be justified by the fact that women expected themselves as achieved the recommended birth spacing intervals and risk free of unintended pregnancy as well as its consequences.

### Limitations of the study

This study has some limitations: the study did not assessed factors related to the health system, service providers and sociocultural related misconception on postpartum sexual activity. Although community based study was conducted to minimize sampling bias, less attention was given for women with severe comorbidities, which may reduce generalizability of study results. In addition, respondents might have limited ability to remember exact time of sexual resumption; and the study lacked detailed information on sexual function and satisfaction. Moreover, evidence on the role of postpartum intercourse in the improvement of sexual health and intimacy between partners was not assessed.

## Conclusion

The study found that more than one fourth of women had resumed sexual intercourse within six weeks of following childbirth. Urban resident, one parity, husband demand for sexual intercourse, use of family planning, and having PNC visit were the significant factors of early resumption of sexual intercourse after childbirth. The finding of this study suggests that health care providers should give postpartum sexual health counseling and interactive health education, particularly for those women with few children and from urban areas. The finding also suggests the need of integrating discussions of postpartum sexual activity into routine prenatal, intrapartum and postnatal care with collaborative effort of policy makers, program planners, health care providers and other stakeholders. Moreover, spousal communication on postpartum sexual activity should be encouraged.

## Data Availability

The datasets used and analyzed during the current study are available from the author.

## Abbreviations

ANC: Antenatal care
AOR: Adjusted odd ratio
CI: Confidence intervals
OR: Odds ratio
PNC: Postnatal care
WHO: World Health Organization

## References

[1] Lamont J (2012). Female sexual health consensus clinical guidelines. J ObstetGynaecol Can.

[2] WHO. WHO Technical Consultation on Postpartum and Postnatal Care. Geneva, Switzerland: World Health Organization; 2010 p 11–16 WHO/MPS/.10.03. http://whqlibdoc.who.int/hq/2010/WHO_MPS_10.03_eng.pdf. 26269861

[3] Organization WH. Postnatal Care for Mothers and Newborns: Highlights from the World Health Organization 2013 Guidelines. In. 2015; 25. 2019.

[4] Radziah MSK, Jamsiah M, Normi M, MohdZahari TH, NurSyimah AT, Nor AM (2013). Early resumption of sexual intercourse and its determinants among postpartum Iban mothers. International Journal of Reproduction, Contraception, Obstetrics and Gynecology Radziah M et al. Int J Reprod Contracept Obstet Gynecol.

[5] Organization WH. Postnatal care for mothers and newborns: Highlights from the World Health Organization 2013 Guidelines. Avaible from: https://www.who.int.maternal.child.adolescent.publications/WHOMCA-PNC-2014-Briefer A. 2015;4.

[6] Leeman L, Rogers R (2012). Współżycie płciowe po porodzie. Aktywność seksualna kobiety po urodzeniu dziecka. Obstet Gynecol.

[7] O'Malley D, Higgins A, Smith V (2015). Postpartum sexual health: a principle-based concept analysis. J Adv Nurs.

[8] Radziah M, Shamsuddin K, Jamsiah M, Normi M, Zahari T, Syimah A (2013). Early resumption of sexual intercourse and its determinants among postpartum Iban mothers. Int J Reprod Contracept Obstet Gynecol.

[9] Borda M, Winfrey W. Postpartum fertility and contraception: an analysis of findings from 17 countries, Baltimore: Jhpiego. Afr J Reprod Health 2010; 14(4 Spec no): 72–79. 2010. 21812200

[10] Woolhouse H, McDonald E, Brown SJ (2014). Changes to sexual and intimate relationships in the postnatal period: women’s experiences with health professionals. Aust J Prim Health.

[11] Organization. WH (2014). Partner release programming strategies for postpartum family planning Global Health. Sci Pract.

[12] Anzaku AMS (2014). Postpartum resumption of sexual activity, Sexual Morbidity and Use of Modern Contraceptive Among Nigerian Women in Jos. Ann Med Health Sci Res.

[13] Kola M, Owonikoko AGA, Aramide M, Tijani (2014). Determinants of resumption of vaginal intercourse in puerperium period in Ogbomoso: consideration for early use of contraceptives. Int J Reprod Contracept Obstet Gynecol.

[14] Mbekenga CKOP, Pembe AB, Draj E, Christensson K (2013). Prolonged sexual abstinence after childbirth: gendered norms and perceived family health risks. Focus group discussions in a Tanzanian suburb. BMC Int Health Hum Rights.

[15] Eliason SK, Bockarie AS, Eliason C (2018). Postpartum fertility behaviours and contraceptive use among women in rural Ghana. Contracept Reprod Med.

[16] Organization WH. Consolidated guideline on sexual and reproductive health and rights of women living with HIV: World Health Organization; 2017. 28737847

[17] Jonas K, Crutzen R, van den Borne B, Reddy P (2017). Healthcare workers’ behaviors and personal determinants associated with providing adequate sexual and reproductive healthcare services in sub-Saharan Africa: a systematic review. BMC Pregnancy Child.

[18] Olsson A, Robertson E, Falk K, Nissen E (2011). Assessing women’s sexual life after childbirth: the role of the postnatal check. Midwifery..

[19] Bureau (ARHB): ARH. Wereda Based plan 2010. Bahir Dar, Ethiopia 2012.

[20] Adenike O-BI, Oke OS, Kola OM, Akande R, Tolulope F, Oluwatosin D, et al. Post-partum resumption of sexual intercourse and the uptake of modern contraceptives among women attending a tertiary hospital in SOUTH West Nigeria. J Health Med Nurs 2017;1(1):65–80. ISSN 2520-4025.

[21] Alum AC, Kizza IB, Osingada CP, Katende G, Kaye DK (2015). Factors associated with early resumption of sexual intercourse among postnatal women in Uganda. Reprod Health.

[22] Fagbamigbe A, Awoyelu I, Akinwale O, Akinwande T, Enitilo B, Bankole O (2018). Factors contributing to the duration of postpartum abstinence among Nigerian women: semi-parametric survival analysis. Heliyon..

[23] Zhuang C, Li T, Li L (2019). Resumption of sexual intercourse post partum and the utilisation of contraceptive methods in China: a cross-sectional study. BMJ Open.

[24] Shabangu Z, Madiba S (2019). The role of culture in maintaining post-partum sexual abstinence of Swazi women. Int J Environ Res Public Health.

[25] Imo CK, Okoronkwo E, Ukoji V, Mbah C (2018). Postpartum sexual abstinence and its implications for under-five health outcome among childbearing women in South-East Nigeria. Afr J Reprod Health.

[26] Ezebialu I, Eke A (2012). Resumption of vaginal intercourse in the early postpartum period: determinants and considerations for child spacing in a Nigerian population. J Obstet Gynaecol.

[27] Iliyasu Z, Galadanci HS, Danlami KM, Salihu HM, Aliyu MH (2018). Correlates of postpartum sexual activity and contraceptive use in Kano, northern Nigeria. Afr J Reprod Health.

[28] Sok C, Sanders JN, Saltzman HM, Turok DK (2016). Sexual behavior, satisfaction, and contraceptive use among postpartum women. J Midwifery Women’s health.

[29] Vaismoradi M, Turunen H, Bondas T (2013). Content analysis and thematic analysis: implications for conducting a qualitative descriptive study. Nurs Health Sci.

[30] Reuben Mahiti G, Mbekenga CK, Dennis Kiwara A, Hurtig A-K, Goicolea I (2017). Perceptions about the cultural practices of male partners during postpartum care in rural Tanzania: a qualitative study. Glob Health Action.

[31] Mbekenga CK, Christensson K, Lugina HI, Olsson P (2011). Joy, struggle and support: postpartum experiences of first-time mothers in a Tanzanian suburb. Women Birth.

[32] Lamont J, Bajzak K, Bouchard C, Burnett M, Byers S, Cohen T (2012). Female sexual health consensus clinical guidelines. J Obstet Gynaecol Can.

[33] Kitessa D, Rulisa S, Ntasumbumuyange D, Aimable M, Pritchett N, Ghebre R. Immediate Postpartum Family Planning Preferences Among Couples in Rwanda. Rwanda Med J. 2019;76(4):1–7.

[34] Osinde MO, Kaye DK, Kakaire O (2012). Influence of HIV infection on women’s resumption of sexual intercourse and use of contraception in the postpartum period in rural Uganda. Int J Gynecol Obstet.

